# Renewed call for action: Highlight negative results to improve science

**DOI:** 10.1016/j.clinsp.2024.100426

**Published:** 2024-06-28

**Authors:** Bo Pei, Huiye Yang, Shixuan Peng

**Affiliations:** a Department of Oncology, Central Hospital of Enshi Tujia and Miao Autonomous Prefecture, Enshi Clinical College of Wuhan University, Enshi, China; b Department of Radiation Oncology and Medical Oncology, Zhongnan Hospital of Wuhan University, Wuhan, China; c Department of Hematology, Affiliated Hospital of Guilin Medical University, Guilin, China; d Department of Oncology, Graduate Collaborative Training Base of The First People's Hospital of Xiangtan City, Hengyang Medical School, University of South China, Hengyang, China

Recently, on March 14, 2024, The New England Journal of Medicine published a negative result in an article entitled “RSV Prefusion F Protein-Based Maternal Vaccine ‒ Preterm Birth and Other Outcomes”.^1^ In the meantime, on August 15, 2023, JAMA also published three consecutive articles with negative findings in the same issue.^2^, ^3^, ^4^ Many researchers have been encouraged by the publication of several articles with negative results in a row. In this scenario, the authors would need to call on more researchers and journals to pay attention to the publication of studies with negative results. Negative results are an important part of science. However, nearly everyone is afraid of negative results. If a study gets negative results, the author is usually very upset and often dares not write an article.

Why is it so hard to publish a negative result? The academic environment is the way it is, and people prefer to see good results. Positive results are preferred in academic publishing, whereas negative results are often less likely to be submitted, accepted, published, or made public. Only a small percentage of journals have publicly declared their willingness to accept negative results for publication. For a long time, the publication of negative results was not well received. It leads to a decline in scientific objectivity and authenticity in published papers. In fact, the academic community has long recognized this problem and has given it a name: “Publication Bias”.

Anyone who has done scientific research knows that there are far more negative results than positive results in experiments, but the number of published papers with negative results is relatively small. Is a negative result meaningless or worthless? Apparently not. In fact, many people think it is necessary to publish the negative results. Scientists have become accustomed to celebrating only successes, forgetting that most technological advances stem from failures. The authors all want to see that these research results can save lives and solve difficult problems in various areas of the world, but I think that too one-sided pursuit of positive results is very dangerous. When experiments with negative results cannot be published in high-impact journals, other researchers cannot learn from them and eventually repeat failed experiments, resulting in a waste of research funds and a lot of time, and delaying the progress of research.

Although a negative study may not solve a scientific problem in a certain field, it points out how other researchers can avoid the same mistakes, which is also important. In addition, the publication of negative results can also guide some practices. For example, there was a rush to buy Intravenous Immunoglobulin (IVIg) in China during the COVID-19 pandemic, which caused an acute shortage of IVIg in a lot of hospitals. However, a later study showed a negative result for IVIg therapy in the treatment of adult COVID-19.^5^ As previous treatment experiences have led to the misuse of IVIg, many patients with other diseases who really need IVIg are unable to take them because of a lack of drugs. The publication of this guiding clinical practice article with negative results changed the behavior of clinicians in time, gave patients the right treatment, and reduced the waste of medical resources. In a similar vein, an article entitled “A Trial of Lopinavir-Ritonavir in Adults Hospitalized with Severe COVID-19” was published online in the New England Journal of Medicine. This randomized controlled trial evaluated the efficacy and safety of lopinavier-ritonavir in the treatment of hospitalized adult patients with severe COVID-19. The results of this study showed that the combination of lopinavir and ritonavir therapy beyond standard care has not shown significant benefits such as accelerated clinical improvement or reduced 28-day mortality compared with standard care alone and may increase adverse events.^6^ These results confirmed that negative research results can help more patients avoid the misuse of drugs and reduce the potential harm of drugs, so as to guide clinical practice.

In general, negative results also have publication significance. The one-sided pursuit of “positive results” may lead researchers to interpret their research results from a non-objective perspective and, in extreme cases, even commit fraud and data manipulation. At present, academic fraud is often reported to be closely related to the excessive pursuit of “positive results”. Moreover, if young scientists, especially students, are guided by the one-sided pursuit of positive results from the beginning of their academic careers, it will be very unfavorable to the progress of science and even lead to long-term stagnation in a certain field. Scientific research is not only a process of constant exploration but also a process of trial and error. Therefore, the occurrence of negative results is normal, and a negative result does not mean that the study has failed. Some negative results obtained through reliable research methods can also provide valuable information, sometimes even more meaningful than positive results. In terms of evidence-based medicine, they are not fundamentally different. Any clinical study with a reasonable design and rigorous management should be given enough attention. An unexpected negative result may give us more insight; it may overturn some common sense; it may avoid over-treatment; and it may redefine interventions. According to the World Health Organization's statement on public disclosure of clinical trial results, “Researchers are obliged to make their findings public, whether negative, inconclusive, or positive, by publishing or making them public in other ways”.

Scientific research is not just about finding significant, positive results. Negative results are also important for scientific research because they help correct misconceptions, avoid repeating ineffective experiments, and save time and resources for future researchers. Therefore, in the current environment where negative results are still not highly valued, *Nature* published Mehta D's call to “Highlight negative results to improve science”,^7^ but the effect still seems unsatisfactory. Thus, we renew our call for an emphasis on the publication of rigorously designed results that address important questions, whether they are negative or positive. Attaching importance to the publication of negative results is conducive to better promoting the progress of science.

## Authors’ contributions

Bo Pei and Huiye Yang: Analyzed the published literature and drafted the manuscript. Shixuan Peng: Reviewed and edited the manuscript. Bo Pei and Huiye Yang: Contributed equally to this work.

## Funding

This work was supported by the Natural Science Foundation of Enshi Tujia and Miao Autonomous Prefecture Government (D20220060) and the Beijing Bethune Public Welfare Foundation (2023-YJ-041-J-005).

## Declaration of competing interest

The authors declare no conflicts of interest.

## Data Availability

Not applicable.
